# A META-ANALYSIS COMPARING EFFICACY OF CONTINUOUS TERBINAFINE WITH INTERMITTENT ITRACONAZOLE FOR TOENAIL ONYCHOMYCOSIS

**DOI:** 10.4103/0019-5154.62746

**Published:** 2010

**Authors:** N A Trivedi, P C Shah

**Affiliations:** *From the Department of Pharmacology, Govt. Medical College, Baroda, Gujarat, India.*

**Keywords:** *Itraconazole*, *onychomycosis*, *terbinafine*

## Abstract

**Background:**

Toenail onychomycosis is a challenge for clinicians to treat. While both Itraconazole and terbinafine have proven to be effective against onychomycosis, very little is known about their comparative efficacy in achieving mycological and clinical cure.

**Aim:**

The purpose of this meta-analysis is to compare the efficacy of continuous terbinafine with intermittent itraconazole in the treatment of toenail onychomycosis.

**Material and Methods:**

all RCTs comparing continuous terbinafine with intermittent itraconazole were identified from PUBMED and BIDS electronic database.

**Results:**

analysis of total eight trials including 1181 patients state that treatment with continuous terbinafine is more likely to produce mycological and clinical cure compared to intermittent itraconazole with odds ratio 2.3(95% CI, 1.7 to 3.0 *P*<0.0001)

**Conclusion:**

though both itraconazole and terbinafine are well tolerated and highly effective drugs, continuous terbinafine is more effective than intermittent itraconazole at achieving mycological cure of toenail onychomycosis

Onychomycosis is one of the most common nail disease and one of the few that is curable.

Systemic treatment for onychomycosis now includes terbinafine, an allylamine that is primarily fungicidal, and itraconazole, a triazole that is primarily fungistatic. Both represent a major therapeutic advancement over griseofulvin in the treatment of this condition.

For toenail infection, terbinafine is usually taken continuously for 12 weeks, whereas itraconazole is taken either continuously or intermittently that is 1 week in 4 weeks for the same period.

Because therapeutic concentration of itraconazole is believed to persist in the nail for a considerable time after systemic treatment is stopped, intermittent therapy with higher daily doses to achieve and maintain therapeutic concentration might be an effective alternative to continuous treatment.

Such intermittent treatment is widely used currently to treat onychomycosis and is claimed to be as effective for this condition as both continuous itraconazole and continuous terbinafine.[1,2]

This meta-analysis compares efficacy of continuous terbinafine and intermittent itraconazole for the treatment of toenail onychomycosis.

We searched Pubmed and BIDS database from 1966 to march 2008 and considered all Randomized controlled trails (RCTs) that evaluated continuous terbinafine v/s intermittent itraconazole for the treatment of toenail onychomycosis.

Our search was limited to the English literature and RCTs only. We included only those trails where diagnosis of toenail onychomycosis was confirmed by culture to establish the presence of dermatophytes. The doses of itraconazole and terbinafine were 400 mg and 250 mg/day, respectively, and minimum and maximum duration of treatment were 12 and 16 weeks, and a follow-up period was 48-72 weeks.

The statistical analysis was performed by the CMA (Comprehensive Meta analysis) version 2 software and odds ratio was calculated by the fixed effect model.

Total eight trials met with all the inclusion and exclusion criteria. Total 1181 patients were included in RCTs comparing 250 mg of continuous terbinafine therapy v/s 400 mg of intermittent itraconazole for the duration of treatment ranging from 12 to 16 weeks.

The primary efficacy criterion was mycological cure rate (MCR) as defined as negative results on microscopy and negative results on fungus culture of samples taken from target toenail at the end of the follow-up period. The secondary efficacy criterion was clinical cure rate as defined as either nail appearing completely normal or ≤10% of nail plate involvement.

Six RCTs also compared adverse events by both the drugs.

Analysis of eight studies comparing intermittent itraconazole with continuous terbinafine for MCR suggests that terbinafine is more likely to cause mycological cure with odds ratio 2.3 (95% CI, 1.7 to 3.0, *P*≤0.0001) compared to itraconazole [Table 1].

**Table 1:** Showing statistical analysis for Mycological cure rate for each study using Odds ratio with 95% CI

Study name	No of patients	Duration of treatment (wks)	Follow up period (wks)	Odds ratio	Lower limit	Upper limit	Z value	*P*-value
Gupta AK, *et al.*	63	12	48	0.51	0.13	2.03	−0.955	0.339
Gupta AK, *et al.*	101	12	62	1.15	0.51	2.57	0.333	0.739
Brautigam M	200	16	48	2.50	1.32	4.77	2.794	0.005
Tosti A, *et al.*	42	16	36	5.33	0.56	51.09	1.452	0.147
Arca E, *et al.*	34	12	36	1.91	0.44	8.35	0.859	0.391
Kejda J	243	12	48	1.06	0.59	1.90	0.186	0.853
E Glyaln, *et al.*	250	12	72	5.02	2.78	9.04	5.364	8.1* 10^−8^
E Glyaln, *et al.*	248	16	72	4.37	2.34	8.18	4.613	3.9* 10^−6^
Summary (Fixed effect model)	Total 1181	12−16	36−72	2.31	1.76	3.03	6.013	1.8* 10^−9^

Figure 1 is a forest plot for the fixed effect model comparing mycological cure rate for different studies. The odds ratio (with CI) for each trial and their average are marked along a vertical line which represents no treatment effect (OR = 1).

**Figure 1:**
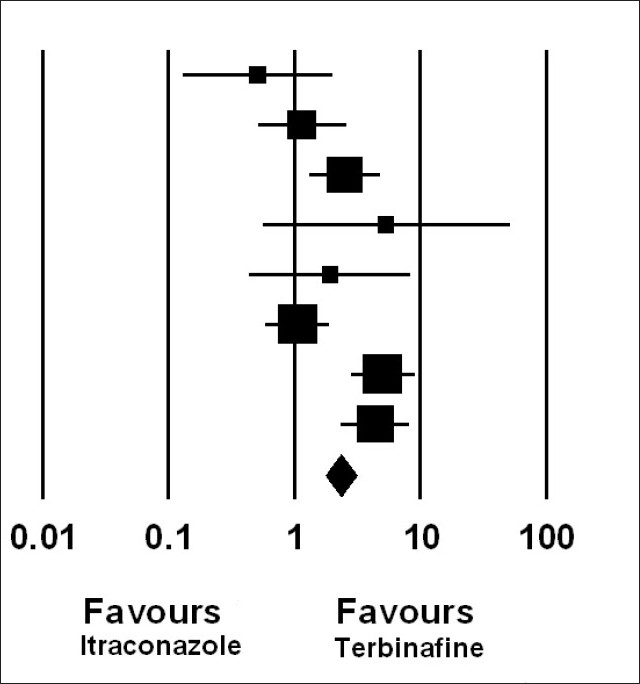
Forest plot with the fixed effect model comparing intermittent itraconazole with continuous terbinafine showing an odds ratio with 95% CI for mycological cure rate

The odds ratio for most studies are on the same side of the vertical line (OR>1), favoring terbinafine, and the test of homogenecity is also highly significant with *P*<0.0001.

Comparing the secondary efficacy end point of clinical cure rate indicates that terbinafine is slightly more likely to produce clinical cure with OR 1.5 (95% CI 1.2 to 2.0 *P*≤0.01) compared to itraconazole.

In all the RCTs except one, ADRs produced by both the drugs were mild to moderate and did not demand treatment discontinuation. The odds ratio for itraconazole with terbinafine for all the trials combined was 1.4 (95% CI, 1.0 to 1.9, P = 0.027) indicating that there was no evidence of overall difference between two treatment effect. However, in one study, 13% patients treated with terbinafine, treatment was discontinued due to ADRs.

In conclusion, both terbinafine and itraconazole are well tolerated and highly effective options for the treatment of toenail onychomycosis, Meta-analysis of the published literatures finds that continuous terbinafine is more effective in achieving mycological cure in comparison to intermittent itraconazole in the treatment of toenail onychomycosis.
